# Flies Avoid Current Atmospheric CO_2_ Concentrations

**DOI:** 10.3389/fphys.2021.646401

**Published:** 2021-04-13

**Authors:** Habibe K. Üçpunar, Ilona C. Grunwald Kadow

**Affiliations:** ^1^Department of Physiology, School of Medicine, Ankara Medipol University, Ankara, Turkey; ^2^TUM School of Life Sciences, Technical University Munich, Freising, Germany

**Keywords:** Drosophila, olfactory system, Gr21a, carbon dioxide, odors, insect

## Abstract

CO_2_ differs from most other odors by being ubiquitously present in the air animals inhale. CO_2_ levels of the atmosphere, however, are subject to change. Depending on the landscape, temperature, and time of the year, CO_2_ levels can change even on shortest time scales. In addition, since the 18th century the CO_2_ baseline keeps increasing due to the intensive fossil fuel usage. However, we do not know whether this change is significant for animals, and if yes whether and how animals adapt to this change. Most insects possess olfactory receptors to detect the gaseous molecule, and CO_2_ is one of the key odorants for insects such as the vinegar fly *Drosophila melanogaster* to find food sources and to warn con-specifics. So far, CO_2_ and its sensory system have been studied in the context of rotting fruit and other CO_2_-emitting sources to investigate flies’ response to significantly elevated levels of CO_2_. However, it has not been addressed whether flies detect and potentially react to atmospheric levels of CO_2_. By using behavioral experiments, here we show that flies can detect atmospheric CO_2_ concentrations and, if given the choice, prefer air with sub-atmospheric levels of the molecule. Blocking the synaptic release from CO_2_ receptor neurons abolishes this choice. Based on electrophysiological recordings, we hypothesize that CO_2_ receptors, similar to ambient temperature receptors, actively sample environmental CO_2_ concentrations close to atmospheric levels. Based on recent findings and our data, we hypothesize that Gr-dependent CO_2_ receptors do not primarily serve as a cue detector to find food sources or avoid danger, instead they function as sensors for preferred environmental conditions.

## Introduction

CO_2_ is released into the atmosphere as a by-product of many natural processes such as organic matter decay or animal metabolic activity. Not surprisingly, many insect species show strong responses to changing CO_2_ stimuli in their environment (Guerenstein and Hildebrand, 2008), and some use elevated levels of CO_2_ as cues for locating food sources (Thom et al., 2004; Dekker and Cardé, 2011), oviposition sites (Stange, 1999), or a sign of danger (Suh et al., 2004). CO_2_ has significant importance for *Drosophila melanogaster* as rotting fruits, the primary food source of this Drosophila species, emit CO_2_. It has been shown that different activity states may induce attractive or aversive responses to elevated levels of CO_2_ in *Drosophila*. While attraction to elevated levels of CO_2_ is mediated by IR25a receptor neurons (van Breugel et al., 2018) in the active, flying state associated with foraging, aversion is mediated by the Gr63a/Gr21a neurons (Suh et al., 2004; Jones et al., 2007). The reason flies avoid CO_2_ remains unclear, however, it has been suggested to help flies avoid dangerous situations by avoiding the odor emitted by groups of stressed flies (aka *Drosophila* stress odor, dSO).

**FIGURE 1:**
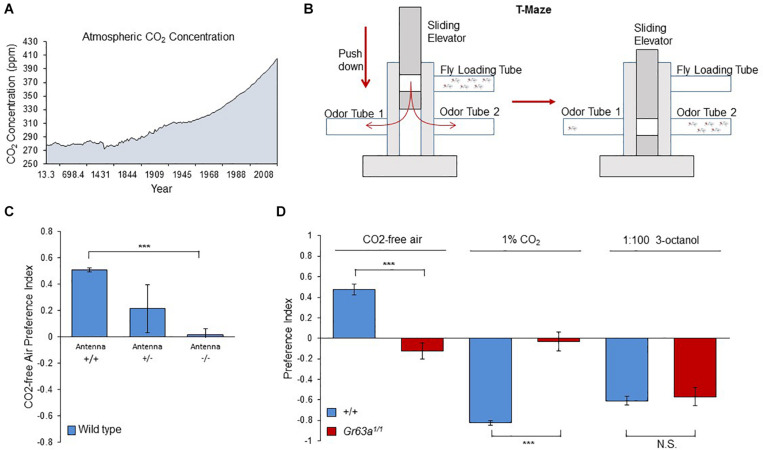
Flies can detect and are attracted to sub-atmospheric CO_2_ concentrations. **(A)** The change of atmospheric CO_2_ concentration as a result of human activities. The graph is drawn by using the merged data of atmospheric CO_2_ record based on ice core data before 1958 (MacFarling Meure et al., 2006) and yearly averages of direct observations from Mauna Loa and the South Pole after and including 1958 (Keeling et al., 2005). **(B)** The olfactory T-maze set-up used for olfactory choice behavior. **(C)** Response of antennaless wild-type flies to CO_2_-free air over the atmospheric air. Antennaless flies are no longer attracted to CO_2_-free air (*n* = 4). **(D)** Response of *gr63a*^1/1^ flies to CO_2_-free air, 1% CO_2_ and 3-octanol. *gr63a*^1/1^ flies showed no attraction to CO_2_-free air and no aversion to CO_2_, while OR-dependent 3-octanol sensitivity is still intact (*p* = 0.0001, *n* = 6–8). Significance assessed by T-test (*p* < 0.001). Error bars represent SEM. (ns > 0.05, *p ≤ 0.05, **p ≤ 0.01, ***p ≤ 0.001).

So far, CO_2_ and its sensory system have been studied in *Drosophila* with relatively high levels of CO_2_ (i.e., 1–5%) with few exceptions (Faucher et al., 2006; Andrea Yao and Carlson, 2010; Bräcker et al., 2013). However, CO_2_ differs from other olfactory cues by being ubiquitously present in the ambient air of the environment of the animal, and its concentration fluctuates throughout the year (Keeling et al., 2005). Moreover, since the 18th century the CO_2_ base line keeps increasing due to the intensive fossil fuel usage, exceeding the 400 ppm (0.04%) threshold in 2014 and reaching 414 ppm in July 2020 (Keeling et al., 2005; Figure 1A). However, it is not known whether insects can detect these atmospheric level changes or even react to them behaviorally. Therefore, we have asked whether a role, or possibly even one of the main roles of the fly CO_2_ receptor is to inform the animal of the ambient concentrations of the gas in its environment allowing them to find their preferred location, similar to ectothermic animals navigating environments of different temperatures (Giraldo et al., 2019). To this end, we gave naïve flies the choice between CO_2_-free air and atmospheric air (400 ppm CO_2_) using the T-maze assay, a two-choice olfactory maze (Figure 1B). Surprisingly, flies showed a strong avoidance of the current atmospheric air and preferred the CO_2_-free side (Figure 1C). We next surgically removed the third segment of the antenna, the main olfactory organ (Vosshall and Stocker, 2007) (Figure 1C), and tested these flies for their preference of CO_2_-free air. Indeed removal of both antennae completely abolished the flies’ choice between atmospheric air and CO_2_-free air (Figure 1C). As shown in previous studies, avoidance of elevated levels of CO_2_ requires co-expression of two gustatory receptors, Gr21a and Gr63a (Jones et al., 2007; Kwon et al., 2007) in olfactory receptor neurons (ORNs) located on those segments of the antenna. Therefore, we tested *gr63a* mutants for their response to CO_2_-free vs. atmospheric air. As expected, these mutants showed no preference between 1% CO_2_ and atmospheric CO_2_ levels compared to controls that strongly avoided it (Figure 1D). In addition to that, *gr63a* mutants also completely lost their reaction to CO_2_-free air as compared to controls that clearly avoided atmospheric levels (Figure 1D). This result demonstrates that the same receptors are used to detect elevated and atmospheric concentrations of CO_2_. Moreover, these results indicate that flies compare differences at even lowest CO_2_ concentrations and are capable of detecting atmospheric CO_2_ levels, which, surprisingly, they appear to find repulsive.

**FIGURE 2:**
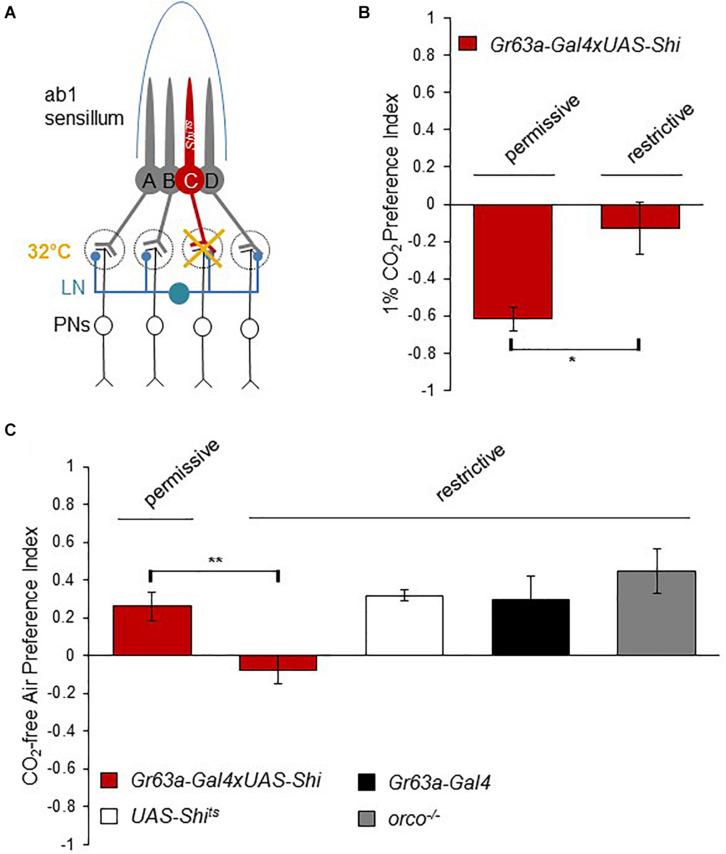
Synaptic release from the CO_2_ neuron is necessary to detect sub-atmospheric CO_2_. **(A)** Neuronal composition of the ab1 sensillum. Neuron C represents the CO_2_ receptor neuron (ab1C) expressing *UAS-shi*^ts1^ under the control of a *Gr63a-Gal4* driver. Synaptic output of ab1C neurons was blocked by shifting flies to 32°C (restrictive) and compared to the behavior of flies tested at 25°C (permissive). **(B)** Response of *Gr63a* > *shi*^ts1^ flies to CO_2_ under permissive and restrictive temperatures. Blocking ab1C neuron output under restrictive temperature reduced CO_2_ avoidance significantly (*p* = 0.025, *n* = 6) compared to permissive controls (*n* = 4). **(C)** Response of *Gr63a* > *shi*^ts1^ flies to CO_2_.-free air at restrictive and permissive temperature. Similar to their loss of aversion to CO_2_, flies under restrictive temperature conditions were not attracted to CO_2_.-free air compared to controls (*p* = 0.0099, *n* = 8). *Gr63a* > *shi*^ts1^ flies under the permissive temperature (*n* = 6) as well as *UAS-shi*^ts1^ (*n* = 8), *Gr63a-Gal4* (*n* = 4), and *orco*^1/1^ (*n* = 8) controls showed attraction to CO_2_.-free air. Significance assessed by T-test. Error bars represent SEM. (ns > 0.05, *p ≤ 0.05, **p ≤ 0.01, ***p ≤ 0.001).

To further understand how flies can distinguish the small concentration differences between atmospheric CO_2_ (400 ppm) and 0 ppm CO_2_, and how the response to CO_2_-free air is processed at the neural level, we next analyzed the requirement of synaptic output from CO_2_ sensory neurons. All olfactory neurons send axonal projections to the first olfactory processing center of the fly brain, the antennal lobe (AL) (Vosshall and Stocker, 2007). CO_2_ sensory neurons project to a particular region of the AL, the V glomerulus. There, they synapse with downstream projection neurons (PNs) that transmit the information to two higher brain centers, the mushroom body and the lateral horn. Additionally, inhibitory as well as excitatory interneurons (LNs) connect glomeruli of different types and likely sharpen the olfactory information content (Wilson, 2013; Figure 2A). Blocking synaptic output of CO_2_ sensory neurons onto downstream neurons has been shown to abolish avoidance of above atmospheric CO_2_ concentrations (Suh et al., 2004). To block synaptic output of CO_2_ sensory neurons, we generated flies that expressed a temperature-sensitive, dominant-negative Dynamin (Shibire, *shi*^ts1^) (Kitamoto, 2001) exclusively in CO_2_ sensory neurons (see Methods). *Shi*^ts1^ blocks synaptic release transiently at temperatures above ∼30°C. We tested *Gr63a* > *shi*^ts1^ flies and controls for their response to CO2-free air in the T-maze at restrictive (32°C) and permissive (25°C) temperatures. Similar to *gr63a* mutants, flies with blocked synaptic output of CO_2_ sensory neurons showed no avoidance of elevated CO_2_ concentrations (Figure 2B) and no preference for CO_2_ -free air compared to controls (Figure 2C). Hence, the presence of the CO_2_ receptor, Gr63a, as well as synaptic output of the CO_2_ sensory neuron are essential for mediating the choice between sub-atmospheric and atmospheric CO_2_ levels. This suggests that CO_2_ perception at atmospheric levels is mediated by the same sensory neuron and the same downstream neurons as used for the detection of much higher CO_2_ concentrations.

**FIGURE 3:**
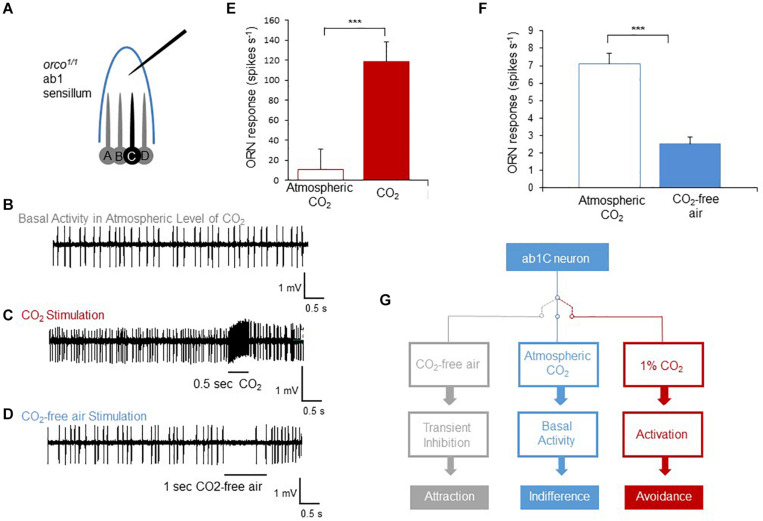
Sub-atmospheric CO_2_ pulses cause silencing of spontaneous activity in CO_2_ receptor neurons. **(A)** Diagram of ab1 single sensillum recording of *orco*^1/1^ flies. A,B, and D neurons cannot respond to stimulation due to the *orco* receptor mutation. **(B–D)** Representative spike traces of spiking activity of ab1C (CO_2_) neurons’ spontaneous activity **(B)**, exposed to 1% CO_2_ **(C)** and exposed to CO_2_-free air **(D)**. **(E–F)** Summary of activity of the ab1C neuron evoked by CO_2_ (*n* = 7) and inhibited by CO_2_-free air (*n* = 8). The number of generated spikes was significantly reduced when ab1C neurons were exposed to CO_2_-free air compared to pre-stimulation activity (*p* < 0.0001, *n* = 8). Significance assessed by T-test. Error bars represent SEM. **(G)** Scheme of three activity states of the CO_2_ neuron and the corresponding behavioral outputs. When the receptor neuron is not exposed to any stimulus, it produces spontaneous spikes resulting in behavioral indifference. Transient inhibition of spontaneous activity by CO_2_-free air generates attraction. In contrast, activation of the neuron and an increase of spike number cause avoidance behavior as observed for elevated levels of CO_2_. (ns > 0.05, *p ≤ 0.05, **p ≤ 0.01, ***p ≤ 0.001).

To elucidate the cellular mechanism of how flies can distinguish atmospheric from sub-atmospheric concentrations of CO_2_ through the Gr21a/Gr63a receptor neurons, we measured the spike frequency of the receptor neurons in extra-cellular single sensilla recordings. CO_2_ receptor, also called ab1C, neurons are housed in sensilla containing four different receptor neurons (ab1A-D, Figure 3A). The other three neurons depend on OR signaling. To isolate the signal of the CO_2_ sensory neuron, we recorded sensilla responses to atmospheric air, CO_2_-free air or 1% CO_2_ in *orco* mutant flies as the *orco* mutation prevents the evoked spiking of OR-dependent neurons. As previously shown (Jones et al., 2007), stimulation of CO_2_ sensory neurons resulted in a significant increase of the number of spikes compared to non-stimulated, presumably spontaneously firing neurons (Figures 3C,E). In contrast to the increase of spiking upon stimulation with CO_2_, stimulation with CO_2_-free air resulted in a transient reduction in firing of the sensory neuron during a 1 second stimulation period as compared to atmospheric air or baseline levels (Figures 3D,F). Upon relief of CO_2_-free air stimulation, spiking immediately returned to baseline levels (Figure 3D). Taken together, we concluded that CO_2_ receptors are not only sensitive to relatively high concentrations of CO_2_ above atmospheric air as previously assumed, but also detect CO_2_ concentration changes at or below current atmospheric levels. While stimulation with elevated CO_2_ concentrations results in increased spiking and strong avoidance behavior, stimulation with CO_2_-free air reduces basal spiking in atmospheric air and leads the fly to avoid atmospheric CO_2_ and instead approach sub-atmospheric CO_2_ environments (Figure 3G).

## Discussion

Evaluation of ambient CO_2_ level is crucial for insects as it may signal various cues useful for survival. In this study, we have shown that (i) *Drosophila* flies avoid current atmospheric levels of CO_2_ and (ii) CO_2_ receptors might be useful for other purposes than finding food sources or communicating with conspecifics (i.e., dSO). Thus, our results suggest a so-far not appreciated, novel ethological function for CO_2_ receptors in the insect olfactory system. Our results show that CO_2_ receptors in the antennae detect CO_2_ concentration at or below current atmospheric CO_2_ levels. We propose that similar to temperature receptors (Barbagallo and Garrity, 2015), CO_2_ receptors actively sample a relative concentration change of an environmentally ubiquitous cue. Thus, both positive and negative deviations from the existing atmospheric concentration are represented by the activity of the CO_2_ receptor neuron. While lower than atmospheric CO_2_ concentrations appear to reduce receptor neuron activity, higher CO_2_ concentrations lead to increased spiking. Accordingly, a relative decrease in firing explains the observed preference for sub-atmospheric CO_2_ levels.

At this point, we do not know whether the (basal) firing rate of the CO_2_ receptor neurons in atmospheric air represents spontaneous or evoked activity. Different from other OSNs, Gr-dependent CO_2_ receptor neurons undergo constant receptor-ligand interaction due to the atmospheric/ambient existence of CO_2_, making it challenging to differentiate stimulation-evoked from spontaneous activity. Spontaneous activity appears to be a characteristic of olfactory receptor neurons in *Drosophila* (Wilson, 2013; Wicher and Miazzi, 2021) and various internal (Andrea Yao and Carlson, 2010) and external (Joseph et al., 2012; Cao et al., 2017) factors can change the rate. For instance, increases in temperature increase the basal firing rate, while decreases in temperature decrease it baseline spiking in insect ORNs (Joseph et al., 2012; Cao et al., 2017). It has also been shown that overexpression of a constitutively active G-protein Gα_q_ counteracts and persistently inhibits both the basal and evoked activity of CO_2_ receptor neurons (Andrea Yao and Carlson, 2010). Similarly, certain odorants can transiently inhibit basal CO_2_ receptor neuron activity driving an opposite behavior than if the receptor neuron is depolarized (Cao et al., 2017). On the other hand, antagonists of CO_2_ and other ORNs have been described which can inhibit activity lastingly and thereby suppress behavior rather than evoking it (Turner and Ray, 2009; Turner et al., 2011). While we cannot pinpoint the exact mechanism at this point, the data presented here suggests that a reduction of the CO_2_ receptor neuron activity observed at atmospheric air is a salient change for the animal sufficient to promote a preference for sub-atmospheric CO_2_ concentrations.

Our data further shows that the attraction to CO_2_-free air, as well as the reduction of atmospheric CO_2_ receptor neuron activity was unaffected in *orco* or *ato* mutants (Supplementary Figure 1) suggesting that evoked activity of other chemosensory neurons is dispensable. Therefore, lateral inhibition, dependent on the activity of neighboring OR neurons within the same sensillum described in a previous *Drosophila* study (Su et al., 2012), is likely not involved.

We also showed that silencing the synaptic output of CO_2_ sensory neurons abolishes the preference for CO_2_-free air over atmospheric air. This argues that synaptic transmission of PNs and/or LNs downstream is essential. Due to the non-linear activation of PNs by sensory neurons, PNs are more sensitive to small changes in presynaptic input when receptor neurons fire at a low rate (Kazama and Wilson, 2008). Thus, at the PN level, a reduction of basal activity by a few spikes could have as strong an effect as the several-fold increase of spiking of the receptor neuron.

It has been shown in a previous study that flies are attracted to CO_2_ in an IR25a dependent manner. How can an odor be both attractive and aversive for the same animal? In this study we have shown that Gr63a/Gr21a CO_2_ detecting neurons mediate preference for CO_2_-free air through a transient reduction of Gr63a/Gr21a ORN firing. Altogether, we propose that flies distribute the CO_2_ detection task into two channels. In the first channel, they use the IR25a ORNs to find food sources and these neurons mediate an attraction when stimulated. On the other hand, in the second channel, *Drosophila* use Gr63a/Gr21a ORNs to find a habitable place, preferably low in its CO_2_ content. Up or down oscillation in the activity of these neurons generate aversion or attraction, respectively. However, how the animal switches between these two channels depending on changing needs (looking for food versus habitation area) still remains unclear. Understanding the circuit mechanism of the Gr-dependent CO_2_ channel and conditional switch to the IR-dependent CO_2_ channel may provide a novel perspective of ethologically relevant decision-making mechanisms.

From the ecological perspective, understanding the CO_2_ detection mechanism of *Drosophila* and its particular preference for low-atmospheric concentrations may provide an entry point for studies on differences between the habitat selections of other *Drosophila* species. Moreover, further research can increase our understanding of whether the atmospheric CO_2_ concentrations have a role in the distribution of both *Drosophila* and other insect species, particularly disease transmitting ones, and may even provide tools to predict the impacts of climate change on the habitat selection of these insects.

## Methods

### Fly Genetics

*D. melanogaster* flies were raised on standard corn meal fly food at controlled light and temperature conditions. The following genotypes were used: Figure 1B: (1) *Gr63a*^1/1^*;* Figure 2C and Supplementary Figure 1: (2) *orco*^1/1^; Figures 3B,C: (2) *Gr63a-Gal4* (3) *UAS-shi*^ts1^ (4) *Gr63a-Gal4/UAS-shi*^ts1^. *atonal* mosaic mutant and mosaic control flies (*eyflp; FRT82B CL/FRT82B ato*^*w*^ and *eyflp; FRT82B CL/FRT82B)* were generated by crossing flies carrying *eyflp; FRT82B CL* to *FRT82B ato*^*w*^ or *FRT82B* flies, respectively. The promoter of the eyeless gene drives the expression of FLP recombinase selectively in the eye-antennal disc.

### Behavior

Flies (*D. melanogaster*) were maintained at 25°C with 60% humidity under a 12 h light:12 h dark cycle except for *shibire* experiments. In *shibire* experiments flies were reared at 18°C and transferred to 25°C after eclosion. CantonS strain was used as wild type. In all behavioral experiments, animals were food-deprived 30 h prior to the experiment and were kept on humidified tissue paper. 6–8-day-old animals were tested in groups of 40–60 in a standard non-aspirated T-maze in red light at the same time of the day. CO_2_ stimulus tubes were prepared by mixing air and pure CO_2_ (Westfalen Gas) through mass flow controllers (Natec sensors). Atmospheric air contained 400 ppm or 0.04% CO_2_. CO_2_-free stimulus tubes were prepared by directly filling them from gas bottles with CO_2_-free air (Westfalen Gas). 3-octanol was diluted in paraffin oil and applied onto filter paper (40 μl) in the test tube. The preference index was calculated by subtracting the number of flies on the air side from the odor side and dividing the result by the total number of flies. All data was analyzed using students T-test and GraphPad Prism software.

### Single Sensillum Recordings

Extracellular recordings of *Drosophila* olfactory sensilla were carried out as described (Hartl et al., 2011). Female flies were recorded at 6–8 days after eclosion. All recordings were performed at the same time of the day. A fly was trapped in a truncated pipette tip with its antenna protruding and mounted on a glass slide. For recording, the antenna was trapped on a coverslip with a glass micropipette. A constant flow of humidified air was provided to the head area of the fly. The reference electrode was placed into the eye. The recording electrode was inserted into the antennal basiconic sensilla containing CO_2_-responsive ab1C neurons. Both the reference and the recording electrodes were filled with 0.01 M KCl. CO_2_-free air and 1% CO_2_ stimulations were carried out by a custom-made odor delivery system (Smartec, Martinsried). Each sensillum was stimulated first with CO_2_-free air for 1 s, and afterward with 1% CO_2_ to confirm neuronal identity with an inter-stimulus interval of at least 60 s. Spontaneous and odor-evoked/inhibited extracellular spikes were recorded using a CV-7B headstage and MultiClamp 700B amplifier (Molecular Devices). The recordings were sampled at 10 kHz, digitized and fed into a computer via Digidata 1440A. The action potential spikes were recorded with Clampex 10.2 software and spike sorting and analysis were done manually with Clampfit 10.2 and MS Excel software, respectively. For the calculation of spikes, spontaneous activity was not subtracted from the odor induced/inhibited spikes. One sensillum was sampled from each animal mounted.

## Data Availability Statement

The raw data supporting the conclusions of this article will be made available by the authors, without undue reservation.

## Author Contributions

HÜ carried out all experiments in this study. HÜ and IG conceived the study, interpreted the results, and wrote the manuscript. Both authors contributed to the article and approved the submitted version.

## Conflict of Interest

The authors declare that the research was conducted in the absence of any commercial or financial relationships that could be construed as a potential conflict of interest.

## Supplementary Material

Supplementary Figure 1 **(A)** Response of *ato-/-* flies to CO_2_-free air. IR mutant *ato-/-* flies did not differ in their response to CO_2_-free air over the atmospheric air (*p* = 0.6701, *n* = 8). **(B)** Response of *orco*^1/1^ flies to CO_2_-free air versus atmospheric air, 1:20 3-octanol and 1:100 3-octanol. While losing their sensitivity to 3-octanol, *orco*^1/1^ flies did not differ in their response to CO_2_-free air over atmospheric air (*p* = 0.2227, *n* = 10). Significance assessed by Student’s t test. Error bars represent SEM.
