# Supplementary figures and images for: Flies Avoid Current Atmospheric CO2 Concentrations
Source: Front Physiol. 2021 Apr 13;12:646401. doi: 10.3389/fphys.2021.646401 (PMC8076854; doi:10.3389/fphys.2021.646401)

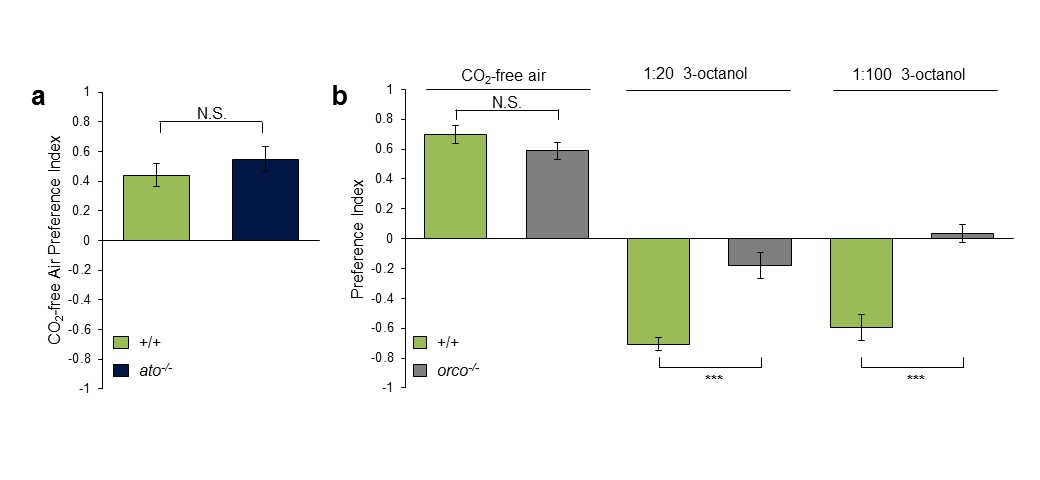

Supplement: Supplementary Figure 1 — (A) Response of ato-/- flies to CO2-free air. IR mutant ato-/- flies did not differ in their response to CO2-free air over the atmospheric air (p = 0.6701, n = 8). (B) Response of orco1/1 flies to CO2-free air versus atmospheric air, 1:20 3-octanol and 1:100 3-octanol. While losing their sensitivity to 3-octanol, orco1/1 flies did not differ in their response to CO2-free air over atmospheric air (p = 0.2227, n = 10). Significance assessed by Student’s t test. Error bars represent SEM. [file Image_1.jpg]
